# Utility of Claudin-3 in extracellular vesicles from human bile as biomarkers of cholangiocarcinoma

**DOI:** 10.1038/s41598-021-81023-y

**Published:** 2021-01-13

**Authors:** Chisaki Ikeda, Hiroaki Haga, Naohiko Makino, Tatsutoshi Inuzuka, Ayako Kurimoto, Toshiki Ueda, Akiko Matsuda, Yasuharu Kakizaki, Tetsuya Ishizawa, Toshikazu Kobayashi, Shinpei Sugahara, Michihiko Tsunoda, Kensei Suda, Yoshiyuki Ueno

**Affiliations:** 1 Department of Gastroenterology, Faculty of Medicine, Yamagata University, 2-2-2 Iidanishi, Yamagata, Yamagata 990-8595 Japan; 2 H.U. Group Research Institute G.K., 51 Komiyamachi, Hachioji, Tokyo 192-0031 Japan

**Keywords:** Biomarkers, Gastroenterology

## Abstract

Extracellular vesicles (EVs) are released from all cells. Bile directly contacts bile duct tumor; bile-derived EVs may contain high concentrations of cancer biomarkers. We performed a proteomic analysis of human bile-derived EVs and identified a novel biomarker of cholangiocarcinoma (CCA). EVs were isolated using ultracentrifugation, and chelating agents, ethylenediaminetetraacetic acid and ethylene glycol tetraacetic acid (EDEG) and phosphate buffered saline (PBS) were used as dissolution solutions. Bile was collected from 10 CCA and 10 choledocholithiasis (stones) cases. Proteomic analysis was performed; subsequently, ELISA was performed using the candidate biomarkers in a verification cohort. The vesicles isolated from bile had a typical size and morphology. The expression of exosome markers was observed. RNA was more abundant in the EDEG group. The proportion of microRNA was higher in the EDEG group. EDEG use resulted in the removal of more contaminants. Proteomic analysis identified 166 proteins as CCA-specific. ELISA for Claudin-3 revealed statistically significant difference. The diagnostic accuracy was AUC 0.945 and sensitivity and specificity were 87.5%. We report the first use of EDEG in the isolation of EVs from human bile and the proteomic analysis of human bile-derived EV-proteins in CCA. Claudin-3 in bile-derived EVs is a useful biomarker for CCA.

## Introduction

Cholangiocarcinoma (CCA) is a tumor originating in the bile duct epithelium and has a poor prognosis. The lack of early clinical symptoms precludes an early diagnosis; consequently, most cases of CCA are diagnosed at an advanced stage^1, 2^. Surgical resection is the only curative treatment for CCA^3^; the median overall survival in unresectable cases is only approximately 6–12 months^4^. Bile duct stenosis is often noted in CCA. However, benign stenosis due to benign diseases, such as primary sclerosing cholangitis, IgG4-related sclerosing cholangitis, secondary sclerosing cholangitis, peribiliary cysts, and Mirizzi syndrome, as well as stenosis due to other unknown origins, may also be observed^5^. Therefore, there may be difficulty in distinguishing benign bile duct stenosis from malignant bile duct stenosis. Although the diagnosis is based on a combination of imaging and histological examination, cytology performed on brush biopsy specimens and bile duct biopsy specimens has a low diagnostic sensitivity of only 20%^6, 7^. Therefore, the identification of novel biomarkers for early diagnosis is important.

In recent years, biomarkers within extracellular vesicles (EVs) have been studied in various diseases. EVs include exosomes, microvesicles, and apoptotic bodies and are 40–1000 nm in size^8^. EVs are secreted from various cells and contain DNA, RNA, proteins, and lipids. Since EVs are surrounded by a lipid bilayer membrane, they can transport their contents in a stable state, even in body fluids. EVs contain molecules derived from the cells that secrete them; therefore, they may be biomarkers specific to certain cell types and cell states^9–11^. A relatively large numbers of EVs are produced by cancer cells where they are involved in the growth and differentiation of tumors^12^; thus, they have been identified as novel biomarkers of cancer. Serum EV-derived proteins, such as aminopeptidase N (AMPN), pantetheinase (VNN1), and polymeric immunoglobulin receptor (PIGR), have been reported as biomarkers of CCA^13^. However, EVs in circulating body fluids, such as blood, reflect all pathophysiological changes that occur in the body. EVs in circulating fluids may also be affected by comorbidities^14^. On the contrary, EVs in non-circulating body fluids such as bile are found less susceptible to events in distant organs. Furthermore, bile is a proximal fluid that comes into direct contact with the CCA itself and is therefore considered to be minimally affected by events in other organs^15^; bile also has high disease specificity suitable for bile duct cancer diagnosis.

Nevertheless, few studies on human bile-derived EVs in CCA have been reported. It could be because the properties and viscosity of bile vary greatly due to bile stasis, inflammation, and infection, which increase the difficulty in isolating pure EVs. Regarding human bile-derived EVs, Li et al. reported the usefulness of a diagnostic panel for microRNA (miRNA) in human bile-derived EVs^15^. The possibility of differentiating benign and malignant bile duct stenosis is based on the concentration of bile-derived EVs^16^.

However, no reports have been published on the search for a protein biomarker of CCA in human bile-derived EVs. Proteins in EVs strongly represent active mechanisms in cancer cells^17^, which make them a good source of potential biomarkers. Therefore, we performed a comprehensive analysis that focused on proteins in human bile-derived EVs.

A problem in analyzing human bile-derived EVs is that the properties of bile are likely to change due to cholestasis, inflammation, and infection, which makes stable collection of pure EVs difficult. Therefore, new methods that allow for the isolation of higher-purity EVs are needed^18^. To efficiently remove contaminants, the utility of a chelating agent, EDEG (ethylenediaminetetraacetic acid and ethylene glycol tetraacetic acid) has already been reported^19^; we believed that this chelating agent could be applied to the isolation of EVs from human bile.

On the contrary, EVs contain cell-derived proteins in a more concentrated and stable state, which results in the easier detection of specific proteins. Therefore, EVs are also considered an ideal biomarker source for proteomic analysis^20^. In reality, however, protein aggregates with similar diameters and proteins such as serum albumin and immunoglobulin are mixed with the protein of interest during EV isolation^17^. These proteins often interfere in detecting trace proteins within EVs that could be biomarkers. For the detection of specific biomarkers, a method that enables the isolation of high-purity EVs with minimal protein contaminants is needed^18^. Presently, no EV separation method specializing for bile has been established.

Thus, this study aims to establish a high-purity isolation method using a chelating agent for human bile-derived EVs and, consequently, by proteomic analysis, identify novel biomarkers for CCA.

## Results

### Clinical characteristics of the patients

The clinical characteristics of the subjects were not significantly different between the CCA and stone groups in terms of age and gender. No significant difference was observed between the two groups in terms of serum CEA and CA19-9 levels. However, alkaline phosphatase (ALP) levels were significantly higher in the CCA group than in the stone group (p = 0.0147; Supplementary Table S1).

In the validation cohort, total bilirubin (T-bil), ALP and gamma-glutamyl transpeptidase (γ-GTP) levels were significantly higher in CCA cases (p = 0.04, 0.01, 0.01; Supplementary Table S2).

### EV isolation from human bile and RNA extraction

**Figure 1:**
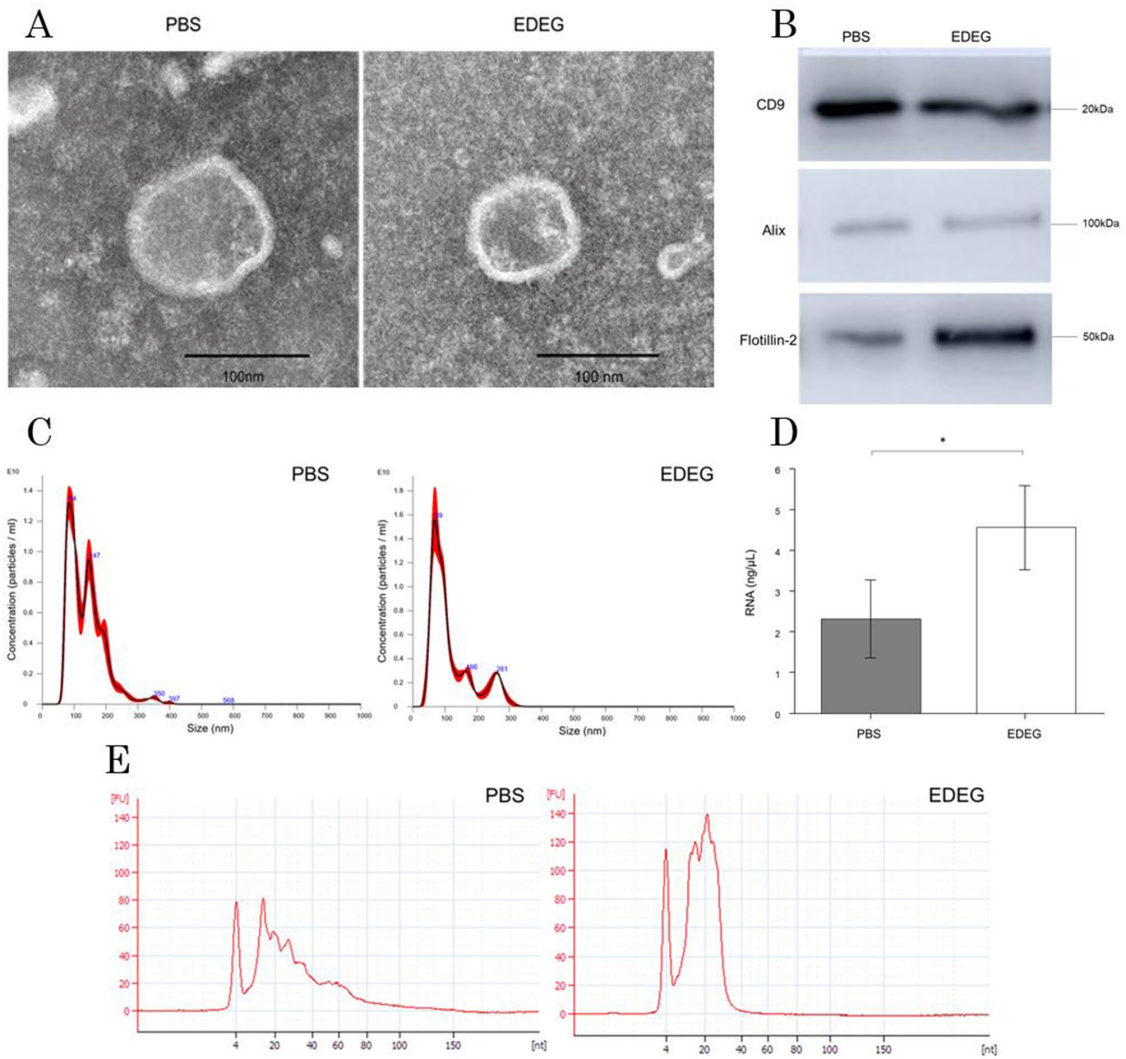
Comparison of the PBS and EDEG groups in EVs. (**A**) TEM image of EVs derived from human bile samples. Both the PBS and EDEG groups contained circular and cup-shaped nanoparticles, which are representative of EVs. The scale bar represents 100 nm. (**B**) Western blot analysis of exosome marker proteins (CD9, ALIX, and Flollitin-2). Exosome marker proteins were detected in both EDEG and PBS groups. (**C**) Nanoparticle tracking analysis of human bile-derived EVs. Representative particle size distribution maps of the PBS and EDEG groups are shown. The particle concentration was 1.92 × 10^12^/mL in the PBS group and 1.93 × 10^12^/mL in the EDEG group. The particle mode was 82.4 nm in the PBS group and 78.9 nm in the EDEG group and was smaller in the EDEG group. (**D**) Comparison of the concentration of RNA extracted from EVs as measured using Qubit 3.0 Fluorometer. The RNA concentration was 2.32 ± 0.96 ng/µL in the PBS group and 4.56 ± 1.03 ng/µL in the EDEG group, which indicated that RNA concentration was significantly higher in the EDEG group. *p < 0.05. (**E**) Bioanalyzer electropherograms of the size distribution of miRNA from EVs. The percentage of microRNA (10–40 nt) out of the small RNA (6–150 nt) detected was 84% in the PBS group and 97% in the EDEG group. The miRNA concentration was 1.96 ng/µL in the PBS group and 3.08 ng/µL in the EDEG group. Lower marker peak was at 4 nt, *FU* fluorescence units.

To confirm the presence of EVs in human bile, EVs were isolated from the bile of patients with bile duct stones, and the analysis described was performed. EVs isolated from bile were imaged using transmission electron microscopy (TEM). Human bile-derived EVs from both the PBS and EDEG groups exhibited a typical cup-shaped and round morphology with a diameter of 40–100 nm (Fig. 1A). Western blot analysis was used to detect the exosome markers CD9, ALIX, and Flotillin-2 in both groups (Fig. 1B). The full-length blots are presented in Supplementary Fig. S1. The particle size and number were measured by NanoSight (n = 4) and are shown in Fig. 1C in the typical particle size distribution chart. Although the average number of particles was 1.92 × 10^12^/mL in the PBS group and 1.93 × 10^12^/mL in the EDEG group, the particle size peak was 82.4 nm in the PBS group and 78.9 nm in the EDEG group, which indicates that smaller particles could be separated in the EDEG group (Fig. 1C).

The concentration of RNA extracted from EVs (n = 5) was measured using a Qubit 3.0 fluorometer and was significantly higher in the EDEG group than the PBS group (EDEG 4.56 ± 1.03 ng/µL, PBS 2.32 ± 0.96 ng/µL, p = 0.0429; Fig. 1D). Moreover, the ratio of miRNA out of the small RNA detected was higher in the EDEG group than in the PBS group (EDEG, 97%; PBS, 84%). The miRNA concentration was 3.75 ng/µL and 2.96 ng/µL in the EDEG and PBS groups, respectively (Fig. 1E).

We also compared the EV isolation method of this study with that of a previous study^21^. According to the NTA, in this study, the highest number of particles was the obtained using the EDEG isolation method. The observed number of particles was 8.11 × 10^9^/mL for EDEG, 6.66 × 10^9^/mL for PBS in this study, and 1.25 × 10^9^/mL for the previously reported method (Supplementary Fig. S2).

### Proteomic analysis of EVs from the PBS and EDEG groups

**Figure 2:**
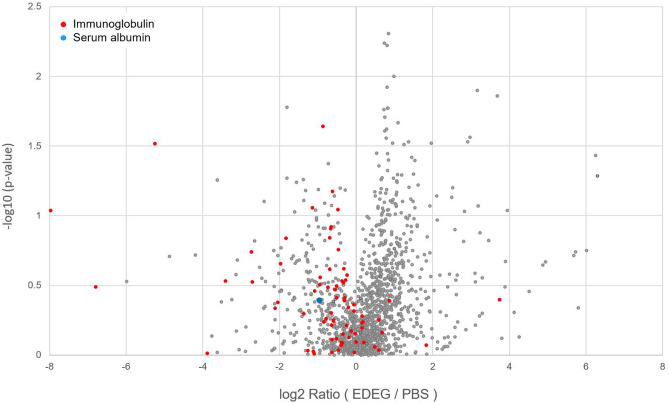
Proteomic analysis of EVs from the PBS and EDEG groups. Volcano plot shows the fold changes and p-values of overlapping proteins in the PBS and EDEG groups. The levels of serum albumin and immunoglobulin were reduced using EDEG. Blue circles indicate serum albumin, and red circles indicate immunoglobulin.

EVs were isolated from the bile of CCA cases (n = 3) using PBS and EDEG, and proteomic analysis was then performed for each group. The detection intensities of each protein were compared between the PBS and EDEG groups, and it was found that the levels of serum albumin and immunoglobulin, which were contaminants, decreased in the EDEG group (Fig. 2).

### Proteomic analysis of CCA and stone cases

**Figure 3:**
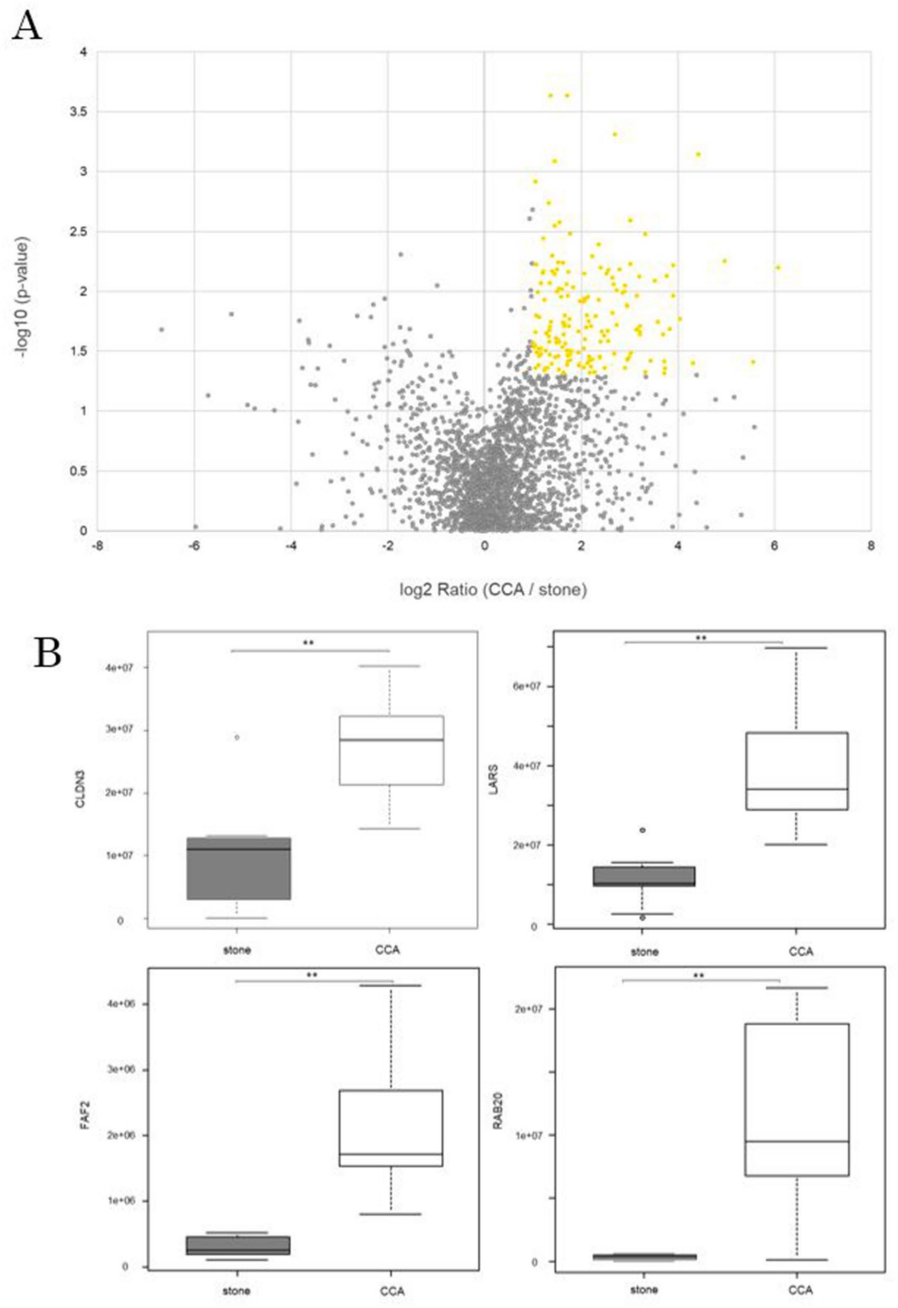
Proteomic analysis of CCA and stone cases. (**A**) The fold change and statistically significant differences in the proteins identified by the proteomic analysis in both cases are shown in the volcano plot. The 166 proteins with a fold change ≥ 2.0 and p < 0.05 that were CCA-specific proteins are indicated by yellow circles. In all, 166 CCA-specific proteins were identified. (**B**) Among the 166 CCA-specific proteins, the top 4 proteins that were statistically significantly different between the stone (gray) and CCA (white) cases are shown in a box plot. The results are displayed as medians, interquartile ranges, minimums, and maximums. The vertical axis indicates the sum of the peak areas of the peptides in the mass spectrum of each detected protein. **p < 0.01.

Bile-derived EVs from CCA (n = 10) and stone cases (n = 10) were separated using EDEG and were further compared by proteomic analysis. The 2350 different proteins identified in both CCA and stone cases were analyzed by volcano plot. In all, 166 proteins were CCA-specific with a fold change ≥ 2.0 and p < 0.05 (Fig. 3A and Supplementary Table S3). Moreover, the expression intensities of the top 4 proteins were compared using a box plot. The p-values of CLDN3, Leucine-tRNA ligase, cytoplasmic (LARS), FAS-associated factor 2 (FAF2), and Ras-related protein rab20 (RAB20) were 0.000232, 0.000233, 0.000491, and 0.000721, respectively (Fig. 3B).

### Diagnostic accuracy of potential biomarker proteins

The concentration of the top 4 CCA-specific proteins in a new validation cohort was measured using ELISA.

**Figure 4:**
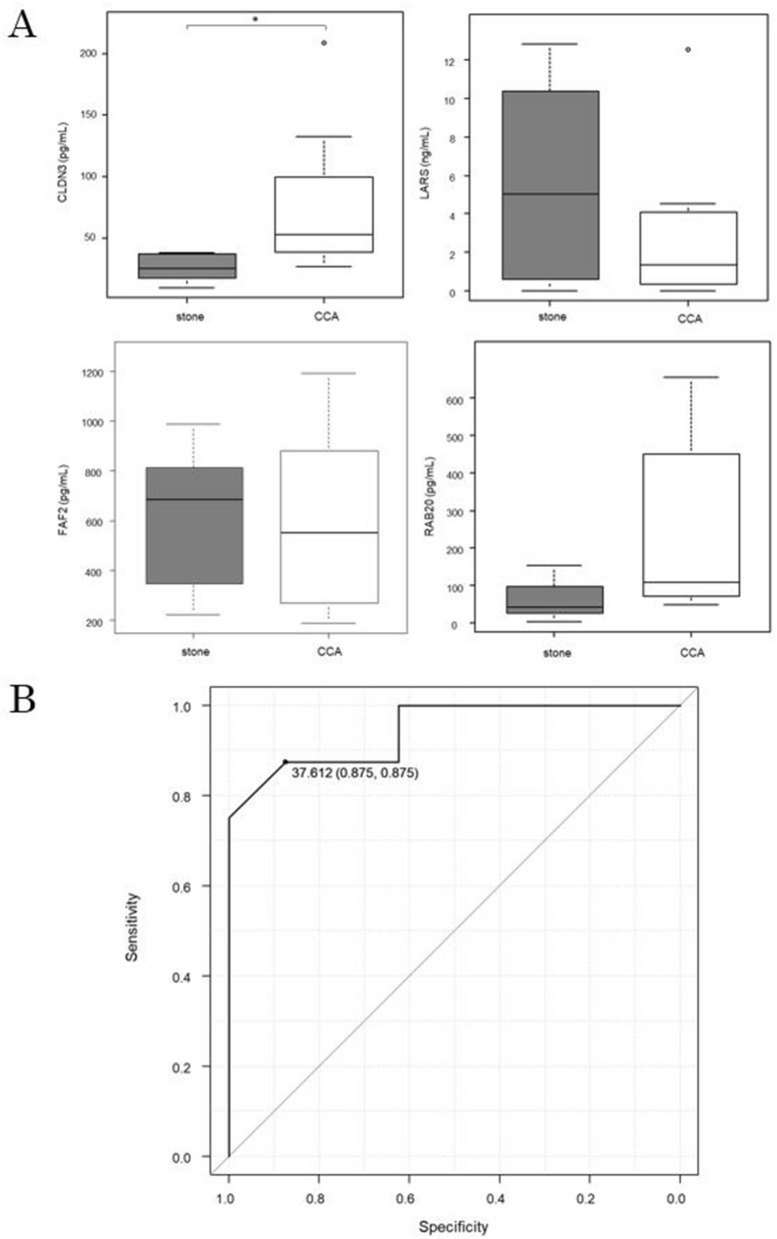
ELISA for the top 4 proteins and diagnostic accuracy of CLDN3. (**A**) Box plots show the concentrations of the top 4 proteins that were significantly differentially expressed in between the stone (gray) and CCA (white) cases, as measured by ELISA. CLDN3 expression was significantly higher in CCA cases. The vertical axis represents the protein concentration. The results are displayed as medians, interquartile ranges, minimums, and maximums. *p < 0.05. (**B**) The ROC curve analysis confirmed that the expression of CLDN3 can be used to discriminate between stones and CCA. At a cutoff value of 37.61 pg/mL, the AUC was 0.945 (95% CI 0.84–1), the sensitivity was 87.5%, and the specificity was 87.5%.

Among the four proteins, only CLDN3 was found to be significantly higher in CCA cases (stone: 25.51 pg/mL, CCA: 76.99 pg/mL, p = 0.0385) (Fig. 4A).

The ROC curve for CLDN3 showed a cutoff value of 37.61 pg/mL, a sensitivity of 87.5%, a specificity of 87.5%, and an AUC of 0.945 (95% confidence interval [CI]: 0.84–1) (Fig. 4B).

### Immunohistochemistry for CLDN3

**Figure 5:**
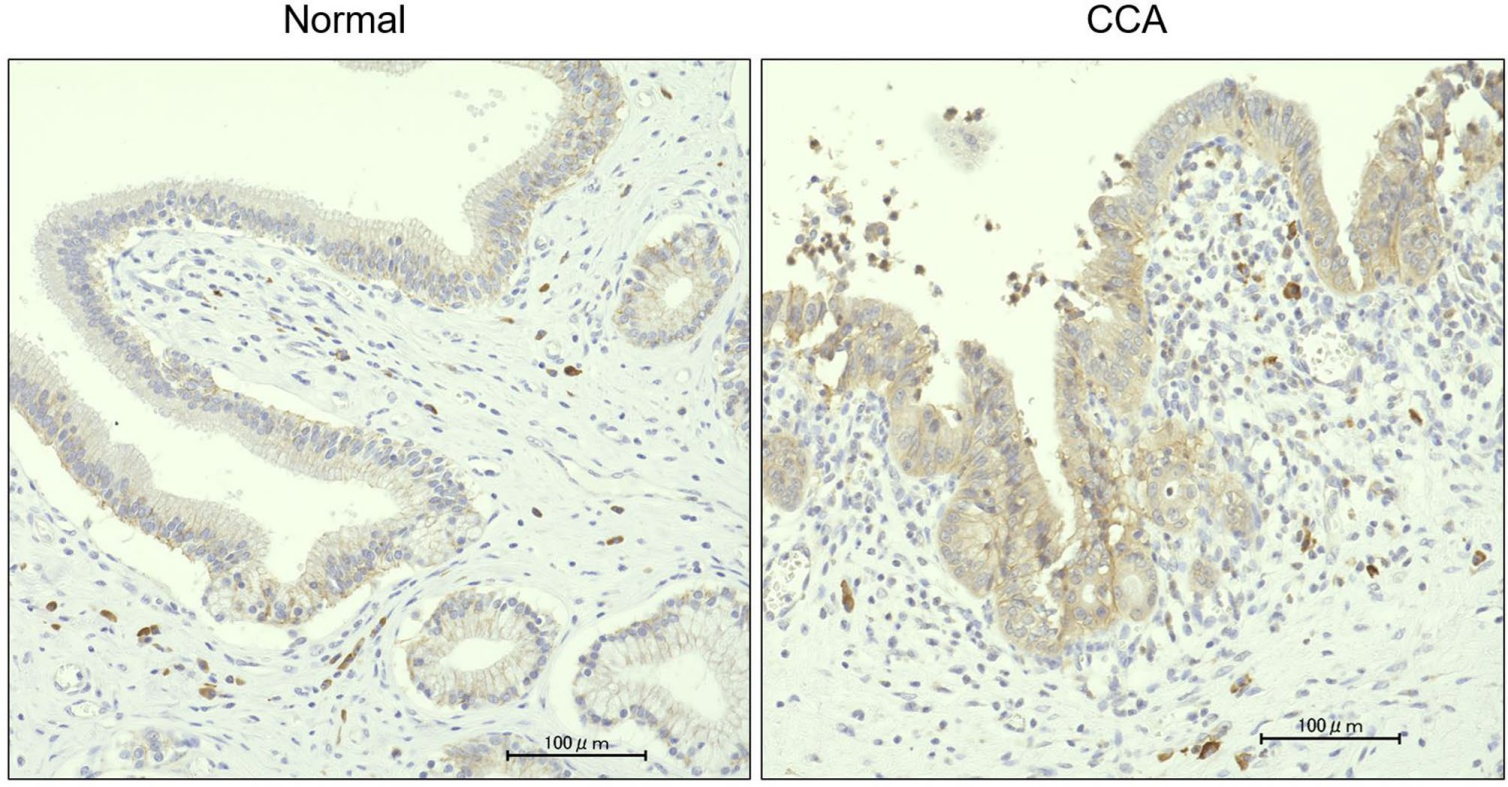
IHC for CLDN3. Immunostaining for CLDN3 in surgical cases of CCA. CLDN3 was strongly expressed in cancer (CCA) compared with normal bile duct epithelium (normal). The scale bar is 100 nm.

We compared CLDN3 staining in surgical specimens obtained from normal bile duct epithelium and carcinoma (n = 5). The cell membrane of normal epithelium was weakly stained, whereas the membrane in carcinoma tissue was found to be strongly stained with a significant difference (Fig. 5 and Supplementary Table S4). Inflammatory cells, such as plasma cells, were stained nonspecifically in all cases.

## Discussion

The most widely used clinical biomarker for cholangiocarcinoma is serum carbohydrate antigen 19-9 (CA19-9). However, CA19-9 may also be elevated in pancreatitis, cholangitis, primary biliary cirrhosis, and in cases of heavy tobacco use^22^.

Bile-derived EVs are highly likely to contain a potential biomarker with high disease specificity. In this study, we have established (1) a method that uses a chelating agent (EDEG) to enable high-purity EV separation from human bile. Furthermore, (2) for the first time, we demonstrated the possibility that CLDN3 in human bile-derived EVs could be a novel biomarker of cholangiocarcinoma.

Although several serum-derived EV proteins have been identified as CCA protein markers^13^, circulating body fluid-derived EVs reflect all pathophysiological changes that occur in the body. Therefore, circulating fluid may be affected by other comorbidities^14^. In contrast, studies using bile samples have suggested the usefulness of a diagnostic panel for bile-derived EV miRNAs. Since bile-derived EV miRNAs are considered more stable in both quality and quantity than whole bile-derived miRNAs, the analysis of the EV inclusion factor is deemed useful^15^. In addition, the difference in concentration of EVs from bile is useful in distinguishing benign and malignant bile duct stenosis^16^. However, it is feared that the concentration of EVs may change easily depending on the degree of obstructive jaundice, the presence of an inflammatory reaction, and the presence or absence of infection. Therefore, in terms of disease specificity, we considered it desirable to target the EV-encapsulating molecules. Furthermore, reproducibility and reliability are important for the development of cancer biomarkers^23^. In other words, the need for a high-purity EV isolation method that minimizes contaminants is a must^24^. In reality, the complexity of human bile and other body fluids^18^ varies greatly. Furthermore, bile contains a large amount of non-specific contaminant proteins. Thus, the problem with human body fluids is that they are difficult to handle compared with culture medium. However, a standardized method for the isolation of EVs from bile has not yet been established.

TEM and Western blot demonstrated that EVs can be isolated using either EDEG or PBS (Fig. 1A,B). Furthermore, when the particle size was measured using nanoparticle tracking analysis (NTA), the EDEG group had a smaller particle size peak than the PBS group, and small particles that were considered to be exosomes were collected (Fig. 1C). When the amount of RNA in the EVs was compared, the RNA concentration was significantly higher in the EDEG group than in the PBS group (Fig. 1D). According to the analysis performed using the bioanalyzer, small RNA was stably present after our EV isolation method and was not degraded. In addition, a large proportion of small RNA was miRNAs (Fig. 1E).

The gold standard for EV isolation is ultracentrifugation^25^. However, since the particle sizes are similar, there is concern that protein aggregates and non-specific proteins may be co-isolated^18^. As a chelating agent, EDEG is useful for EV isolation by immunoprecipitation using an anti-CD9 antibody. EDEG has been shown to facilitate the detachment of proteins that adhere to EVs via metal ions and to remove additional contaminants^19^. In this study, we used this chelating agent as part of the ultracentrifugation method for high-purity EV isolation from human bile. A proteomic analysis has showed that serum albumin and immunoglobulin were reduced in the EDEG group (Fig. 2). This finding suggested that EDEG could be used to reduce contaminants in the isolation of high-purity EVs.

Next, we performed a comprehensive analysis of human bile-derived EV proteins. When all 2350 proteins detected in both CCA and stone cases were compared, 166 proteins were CCA-specific with a fold change ≥ 2.0 and p < 0.05 (Fig. 3A and Supplementary Table S3). CLDN3 was determined to be the most statistically significant protein among all the detected proteins (Supplementary Table S3). To verify the accuracy of CLDN3 for the diagnosis of CCA, we performed an ELISA in a new verification cohort. The CLDN3 concentration was 76.99 pg/mL in CCA cases and 25.51 pg/mL in stone cases; this difference was significantly different (Fig. 4A). According to the ROC curve, the cutoff value was 37.61 pg/mL, the AUC was 0.945 (95% CI 0.84–1), the sensitivity was 87.5%, and the specificity was 87.5%. This suggested that CLDN3 in bile-derived EVs could be a diagnostic marker for CCA (Fig. 4B). Furthermore, CLDN3 immunostaining in surgical specimens showed a higher expression with a significant difference in CCA than in normal bile duct epithelium (Fig. 5 and Supplementary Table S4).

CLDN3 belongs to a protein family that is important in tight junction formation and function. It has been widely accepted that the loss of tight junction function is correlated with cancer progression and metastasis, which suggests that Claudin may be involved in cancer cell survival and invasion^26^. Additionally, the Claudin protein interacts with a number of PDZ domain-containing proteins such as ZO proteins^27, 28^. Many other cytosolic and nuclear proteins, tumor suppressors, and transcription factors have also been shown to interact with tight junction complexes. These interactions can serve an adaptor function for other proteins involved in cell signaling^29–33^. Thus, it has been suggested that tight junctions may play important roles in the regulation functions such as proliferation and tumor suppression. CLDN3 and CLDN4 have been reported to be frequently upregulated in ovarian, breast, prostate, and pancreatic cancers^34^.

Although high expression of claudin-3 in cancer has been reported, few records of detailed values of sensitivity and specificity of biomarkers are available. Diagnostic ability in breast and prostate cancer was assessed using ROC curves. In breast cancer, BRCA mutations were diagnosed by combining immunostaining images of several proteins, including CLDN3 (AUC = 0.946)^35^. Additionally, plasma CLDN3 for prostate cancer was predicted to have a Gleason score ≥ 8 (AUC = 0.705)^36^.

However, their expression patterns and biological functions are largely unknown in human CCA. In this study, CLDN3 was highly expressed in CCA as in other tumor types, which suggests that overexpression of CLDN3 may be involved in cancer survival.

The method established in this study for isolating high-purity EVs from bile is useful for future biomarker studies of human bile-derived EVs and is considered to have high clinical significance. Since ERCP is found to be essential for the diagnosis and treatment of biliary stenosis, we can secondarily collect bile for EV isolation. The simple identification of a novel molecular marker in bile will enable a non-invasive diagnosis rather than the invasiveness necessary for histopathological examination. CLDN3 in bile-derived EVs for CCA has a sensitivity of 87.5% and a specificity of 87.5%. The finding that CLDN3 can be measured by ELISA indicates that CLDN3 might be a useful biomarker for CCA in actual clinical practice.

However, it should be noted that our study has limitations; these are as follows: small number of cases and its single-center design. Moreover, since only stone cases as controls were compared with CCA, comparative studies with other benign bile duct diseases are also desired. We believe that it is important to investigate the bile of benign bile duct stenosis cases in the future using the EV isolation method established in the current study. In addition, it is necessary to examine the possibility of early detection and comparative examination for each stage. Larger studies will be needed in the future to verify the results of this study.

In conclusion, using EDEG enabled the isolation of high-purity EVs from human bile. Furthermore, it was shown that CLDN3 in human bile-derived EVs could be a biomarker of CCA.

## Methods

### Patients and clinical samples

Between October 2017 and January 2019, bile was collected from 10 patients (hilar = 4, distal = 6) who underwent endoscopic retrograde cholangiopancreatography (ERCP) for the diagnosis and drainage of CCA at Yamagata University Hospital. In addition, 10 patients with common bile duct stones were included as a control group (Supplementary Table S1). CCA was histopathologically diagnosed by two pathologists. Pathologic tumor staging was performed in accordance with the 8th Edition of the UICC classification. Bile was collected using a nasal biliary drainage tube (QuickPlace V 6Fr, Olympus, Tokyo, Japan) while being cooled on ice. Samples were snap-frozen at − 80 °C. The validation cohort consisted of 8 CCA cases (hilar = 4, distal = 4) and 8 bile duct stone cases (Supplementary Table S2). Written informed consent was obtained from all participants. This study was performed according to the guidelines described in the Declaration of Helsinki for biomedical research involving human subjects. The study protocol was approved by the institutional review board of the Ethical Review Committee of Yamagata University Faculty of Medicine (Approval number: 2018-12).

### EV isolation

**Figure 6:**
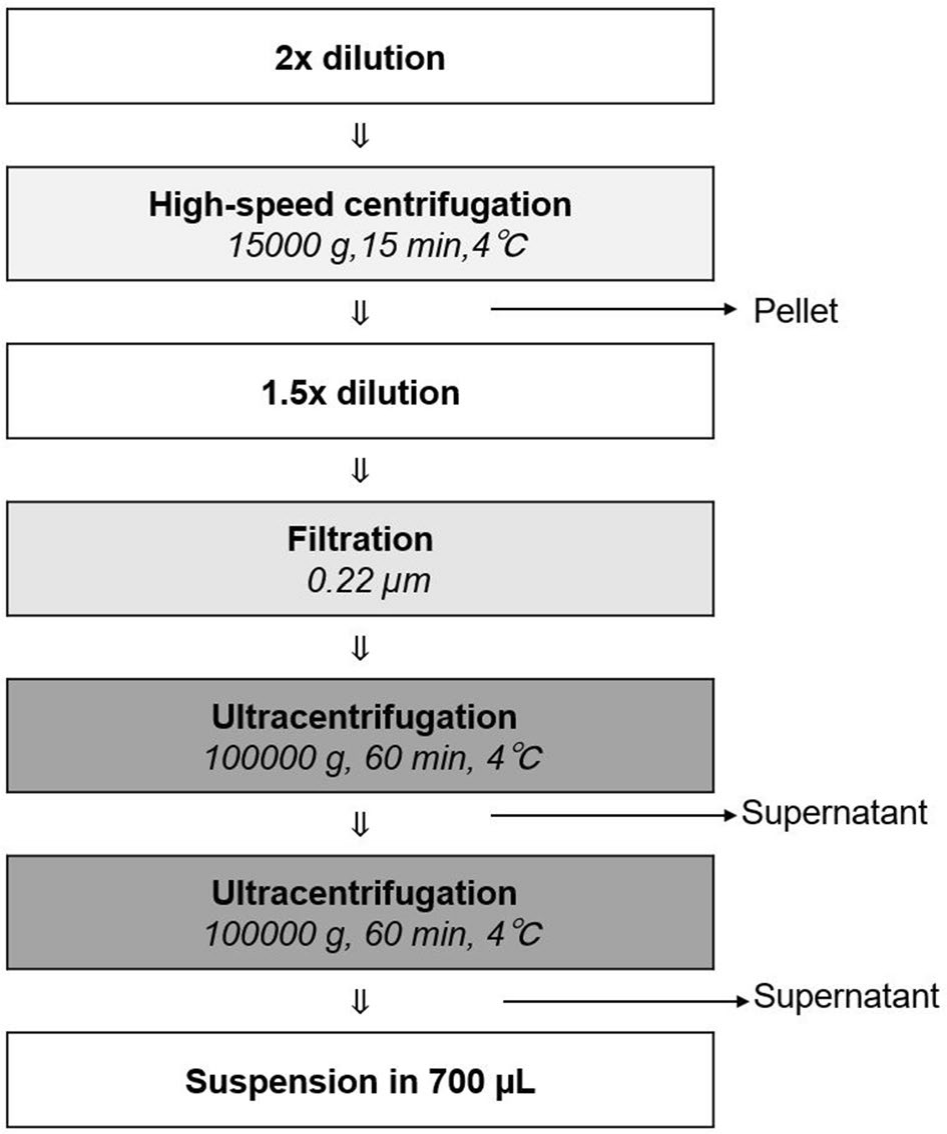
EV isolation method from human bile. The procedure shown below was performed to efficiently isolate high-purity EVs from human bile. EDEG and PBS were used for dilution and suspension. First, to compensate for the viscosity of bile, we performed a two-step dilution. Then, to further remove impurities, high-speed centrifugation and filtration were performed, with ultracentrifugation repeated.

About 400 µL of each bile sample was diluted with the same volume of EDEG or PBS and incubated at room temperature for 10 min. Samples were then centrifuged at 15,000×*g* for 15 min at 4 °C (Tomy MX-307; Tomy Digital Biology, Tokyo, Japan). The supernatant was collected and diluted 1.5 times with EDEG or PBS. The mixture was then filtered through a 0.22-µm filter (Millipore, Billerica, MA, USA). The supernatant was ultracentrifuged at 100,000×*g* for 60 min at 4 °C (himac, Koki Holdings Co., Ltd., Tokyo, Japan, Rotor S52ST-212). The supernatant was then discarded; the resultant pellets were resuspended in 700 µL EDEG or PBS. The samples were ultracentrifuged again after which the supernatant was discarded (Fig. 6). Pellets were stored at 4 °C.

### Transmission electron microscopy (TEM)

For TEM, the EV-containing samples were absorbed into carbon-coated copper grids and stained with a 2% phosphotungstic acid solution (pH 7.0) for 10 s. The grids were observed by TEM (JEM-1400 Plus; JEO L Ltd., Tokyo, Japan) at an acceleration voltage of 100 kV. Digital images (3296 × 2472 pixels) were obtained using a CCD camera (EM-14830RUBY2; JEOL Ltd., Tokyo, Japan).

### Western blotting

The antibodies against CD9 (prepared at H.U. Group Research Institute; 1 µg/mL)^19^, ALIX (1:1000, rabbit polyclonal antibody, Merck Millipore, MA, USA), and Flotillin-2 (1:5000, mouse monoclonal antibody, BD Biosciences NJ, USA) were used.

4 × Laemmli Sample Buffer (Bio-Rad, Hercules, CA, USA) was diluted to 1 ×; 50 µL was used to dissolve the EVs. For ALIX and Flotillin-2 detection, DTT (FUJIFILM Wako Pure Chemical Corporation, Osaka, Japan) was added so that the concentration was 0.1 M. Proteins were separated using Mini-PROTEAN TGX Precast Gels (4–20%, Bio-Rad) and subsequently transferred to polyvinylidene difluoride (PVDF) membranes (ATTO Corporation, Tokyo, Japan). Next, the membranes were blocked at room temperature with 1% skim milk/0.05% Tween-20/PBS overnight. Subsequently, the membranes were exposed to Can Get Signal Immunoreaction Enhancer Solution Solution1 (TOYOBO, Osaka, Japan) and incubated with the primary antibodies for 1 h at room temperature. After incubation, the membranes were then washed. Then, the membranes were incubated with the appropriate secondary antibodies, which included anti-rabbit IgG (Agilent Technologies/Dako, Carpinteria, CA, USA; 0.087 µg/mL) and anti-mouse IgG (Agilent Technologies/Dako; 0.33 µg/mL), for 1 h at room temperature. After a final wash step, the chemiluminescence substrate ECL Select Western Blotting Detection Reagent (GE Healthcare Life Sciences, Bucks, UK) was added to the membranes. Imaging was performed using an ImageQuant LAS 500 imager (GE Healthcare Life Sciences).

### Nanoparticle tracking analysis (NTA)

Vesicle size and particle counts were analyzed using a NanoSight NS300 system (NanoSight, Malvern Instruments, Malvern, UK).

Some of the bile-derived EV pellets were appropriately diluted in PBS.

The Brownian motion of the particles in solution was recorded thrice for 60 s each time; size and number of particles were analyzed using NTA 3.1 software (https://www.malvernpanalytical.com/en/learn/events-and-training/webinars/W150326NanoSightSoftwareRelease).

### RNA extraction, quantification, and qualitative analysis

MicroRNA was extracted from 200 µL EVs using the miRNeasy serum/plasma kit (Qiagen, Hilden, Germany) following the manufacturer’s instructions. The RNA concentration was measured using a microRNA assay kit with a Qubit 3.0 Fluorometer (Thermo Fisher Scientific, Waltham, MA., USA). The quality of the small RNA was assessed using an Agilent 2100 Bioanalyzer and an Agilent Small RNA kit (Agilent Technologies, Santa Clara, CA, USA). Electropherograms were visualized using Agilent 2100 Expert software (Agilent Technologies).

### Preparation of peptide

EVs isolated from bile were lysed in 40 µL of 2% RapiGest SF (Waters, MA, USA) in 50 mM ammonium bicarbonate (Honeywell Fluka, Thermo Fisher Scientific), supplemented with 50 mM DTT (FUJIFILM Wako Pure Chemical Corporation) and incubated at 60 °C for 30 min. The samples were then cooled to room temperature; 4 µL of 150 mM 2-iodoacetamide (Nacalai Tesque, Kyoto, Japan) was added to each sample after which the samples were incubated at room temperature for 30 min in the dark. The lysates were incubated with 1 µg/mL of Mass Spec Grade Trypsin/Lys-C Mix (Promega, WI, USA) at 37 °C overnight. Next a 4 µL of 10% TFA was added to the digested mixture, which was incubated at 37 °C for 30 min. After centrifugation at 13,000×*g* for 10 min at room temperature, the supernatant was lyophilized in a miVac system (Genevac Ltd, Ipswich, UK) and desalted using Pierce C-18 Spin Columns (Thermo Fisher Scientific) according to the manufacturer’s instructions. The resultant peptide was eluted in 70% acetonitrile and was then lyophilized using a miVac system and stored at − 80 °C.

### LC–MS/MS analysis

The resultant peptides were reconstituted in 20 µL of water containing 0.1% formic acid (FA) (Fisher Chemical, MA, USA). The proteomic analysis of the peptides was performed using a Q Exactive Orbitrap mass spectrometer (Thermo Fisher Scientific) equipped with an UltiMate 3000 Nano LC System (Thermo Fisher Scientific). A 1 µL sample was injected into an Acclaim PepMap 1000 trap column (75 µm × 2 cm, nanoViper C18 3 µm, 100 Å, Thermo Fisher Scientific), which was heated to 40 °C in a chamber and then placed in a C18 reverse-phase Aurora UHPLC Emitter Column with nanoZero and Captive Spray Insert (75 µm × 25 cm, 1.6 µm, 120 Å, Ion Opticks Pty Ltd, Victoria, Australia) using the DreamSpray interface (AMR Inc., Tokyo, Japan). The nano pump flow rate was set to 250 nL/min with a 170 min gradient, where the mobile phases were A (0.1% FA in water, Fisher Chemical) and B (0.1% FA in acetonitrile, Fisher Chemical). The gradient was 0–8 min at 2% of B and 8–15 min at 2–15% of B; the gradient was increased to 40% at 149 min and 40%–95% of B at 149–150 min, after which an 8 min wash step was performed, followed by equilibrium for 11 min. Mass spectrometry parameters and those of the Proteome Discoverer 2.2.0.388 software (Thermo Fisher Scientific) (https://www.thermofisher.com/order/catalog/product/OPTON-30810#/OPTON-30810) were described in a previous report^19^.

### Label-free quantification (LFQ) protein analysis

EV proteins were identified in three runs and were quantified with LFQ values using Proteome Discoverer. LFQ parameters were set to Normalization mode: total peptide amount, imputation mode: low abundance resampling, ratio calculation: summed abundance-based. The median of the normalized abundances that were obtained for each identified protein, the median abundances of proteins between the two groups, and the log2 Ratio, –log10 were calculated and were visualized in volcano plots.

### LC–MS/MS data availability

The LC–MS/MS raw data and result files have been deposited with the ProteomeXchange Consortium (http://www.proteomexchange.org/) via the jPOST partner repository^37^ with the data set identifier PXD020349.

### ELISA

The concentration of the four candidate proteins was determined in the validation cohort using each ELISA kit: Human Claudin-3 (CLDN3) ELISA kit (Wuhan Huamei Biotech, Hubei, China), Human Leucine-tRNA ligase, cytoplasmic (LARS) ELISA kit (MyBioSource, CA, USA), Human FAS-associated factor 2 (FAF2) ELISA kit (LifeSpan BioSciences, WA, USA), and Human Ras-related protein rab20 (RAB20) ELISA kit (MyBioSource).

EVs were suspended in 45 µL EDEG and treated with 0.2% of Triton X-100 for 15 min. Then, 450 µL PBS was added to the treated EVs. The levels of proteins were measured according to the manufacturer’s instructions.

For each plate, UV absorption was measured at 450 nm using a Benchmark Plus Microplate Spectrophotometer (Bio-Rad).

### Immunohistochemistry

Immunohistochemistry (IHC) was performed using an antibody against CLDN3 (Abcam, rabbit polyclonal antibody, Cambridge, MA, USA). PBS was used instead of the primary antibody, served as a negative control. Thick sections of 3 µm were then deparaffinized. Antigen retrieval was performed using citric acid in an autoclave. Endogenous peroxidase activity was blocked with PBS containing 0.3% hydrogen peroxide for 15 min at room temperature. Sections were incubated with the primary antibody at 4 °C overnight. The labeled streptavidin–biotin peroxidase method (UltraTech HRP Streptavidin–Biotin Detection system, PN IM2391; Immunotech, Marseille, France) was used. Brown color due to 3,3′-diaminobenzidine tetrahydrochloride (Dojindo, Kumamoto, Japan) indicated a positive reaction.

### IHC analysis

CLDN3 staining was scored by the pathologist in all cases. An IHC score was assigned to each score, as per the reports in a previous study^38^: a product of staining intensity (scale of 0–3) and percentage of tumor cell staining (scale of 0–4).

Staining intensity was scored as 0 (none or minimal staining in occasional tumor cells), 1 (weak), 2 (moderate), or 3 (strong). The percentage of tumor cell staining was scored as 0 (< 1%), 1 (> 0–25), 2 (> 25–50), 3 (> 50–75), or 4 (> 75–100) based on the number of stained cells.

### Statistical analysis

To assess differences between two groups, Student’s *t* test, Mann–Whitney *U* test or Fisher’s exact test was performed. All data are presented as the mean ± SE unless stated otherwise. All statistical analyses were performed with EZR (version 1.40)^39^. P < 0.05 was considered statistically significant.

### Supplementary Information

Supplementary Information.

## Data Availability

The datasets generated during and/or analyzed during the current study are available from the corresponding author on reasonable request.
