# Supplementary material for: Utility of Claudin-3 in extracellular vesicles from human bile as biomarkers of cholangiocarcinoma
Source: Sci Rep. 2021 Jan 13;11:1195. doi: 10.1038/s41598-021-81023-y (PMC7807063; doi:10.1038/s41598-021-81023-y)
Supplement: Supplementary file 1 — Supplementary Information. [file 41598_2021_81023_MOESM1_ESM.pdf]

Utility of Claudin-3 in extracellular vesicles from human bile as biomarkers of cholangiocarcinoma

Chisaki IKEDA[1)], Hiroaki HAGA[1) *], Naohiko MAKINO[1)], Tatsutoshi INUZUKA[2)], Ayako KURIMOTO[2)], Toshiki UEDA[2)], Akiko MATSUDA[1)], Yasuharu KAKIZAKI[1)], Tetsuya ISHIZAWA[1)], Toshikazu KOBAYASHI[1)], Shinpei SUGAHARA[1)], Michihiko TSUNODA[1)], Kensei SUDA[1)], and Yoshiyuki UENO[1)]

1) Department of Gastroenterology, Faculty of Medicine, Yamagata University, 2-2-2 Iidanishi, Yamagata-shi, Yamagata 990-8595, Japan
2) H.U. Group Research Institute G.K., 51 Komiyamachi, Hachioji, Tokyo 192-0031, Japan

Supplementary figure S1. Full-length blots of exosome marker proteins

Western blotting showed that EVs could be isolated in both PBS and EDEG groups. For clarity, the blot for each marker in Case 2 was cut and shown in Figure 1B.

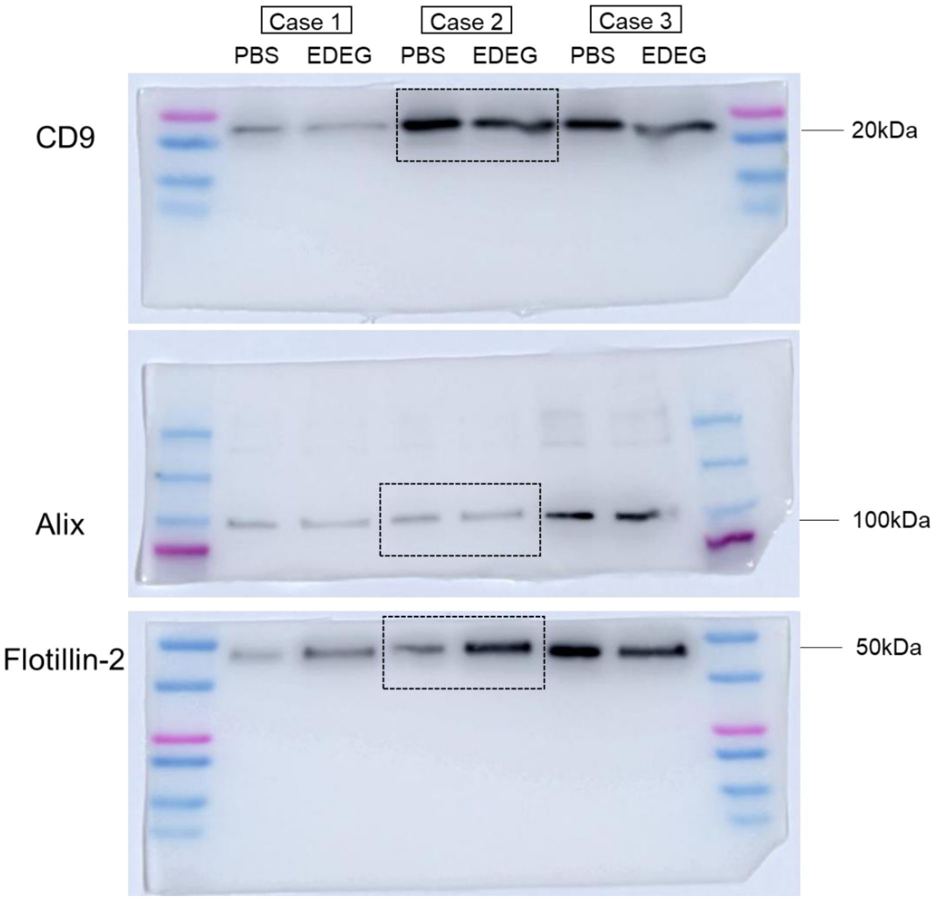

Supplementary figure S2. Comparison of EV isolation methods by NTA

The NTA showed that the isolation method in this study using EDGE had the largest number of particles compared to PBS and previously reported methods.

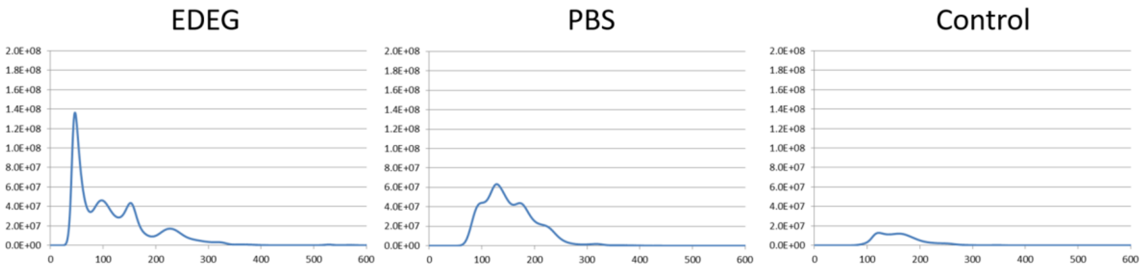

Supplementary Table S1. Characteristics of CCA and stone cases

| | | CCA (n = 10) | | Stone (n = 10) | | P-value | |
|---|---|---|---|---|---|---|---|
| Age | | 78 | (66–95) | 79 | (41–88) | N.S. | |
| Sex (male/female) | | 4/6 | | 7/3 | | | |
| Height | (cm) | 143.0 | (138.5–169.9) | 150.8 | (143.8–178.3) | N.S. | |
| Weight | (kg) | 54.2 | (38.7–68.1) | 59.1 | (40.4–90.3) | N.S. | |
| BMI | (kg/m$^2$) | 23.0 | (16.2–29.0) | 22.9 | (16.6–29.3) | N.S. | |
| | | | | | | | |
| T-bil | (mg/dL) | 9.15 | (0.9–22.6) | 2.35 | (0.5–5.7) | N.S. | |
| AST | (U/L) | 160 | (59–653) | 138 | (17–1536) | N.S. | |
| ALT | (U/L) | 191.0 | (32–912) | 185.5 | (8–1216) | N.S. | |
| ALP | (U/L) | 1274 | (208–2997) | 561 | (136–1086) | 0.0147 | * |
| LDH | (U/L) | 285 | (199–363) | 213 | (115–1469) | N.S. | |
| γ-GTP | (U/L) | 641 | (150–2900) | 529 | (24–885) | N.S. | |
| WBC | (/μL) | 6160 | (3700–7940) | 6435 | (3050–16900) | N.S. | |
| CRP | (mg/dL) | 0.57 | (0–3.14) | 0.51 | (0–22.50) | N.S. | |
| | | | | | | | |
| CEA | (ng/mL) | 2.17 | (1.13–6.23) | 1.45 | (1.04–4.40) | N.S. | |
| CA19-9 | (U/mL) | 450.1 | (6.9–12000.0) | 26.2 | (0–1829.0) | N.S. | |
| | | | | | | | |
| Tumor location | | | | | | | |
| Hilar/distal | | 4/6 | | | | | |
| TNM stage | | | | | | | |
| 1/2/3/4 | | 3/3/3/1 | | | | | |

Median (range)

N.S., not significant

Supplementary Table S2. Characteristics of CCA and stone cases in the validation cohort

| | | CCA (n＝8) | | Stone (n＝8) | | P-value | |
|---|---|---|---|---|---|---|---|
| Age | | 76.5 | (64–93) | 76.5 | (60–89) | N.S. | |
| Sex (male/female) | | 7 / 1 | | 3 / 5 | | N.S. | |
| Height | (cm) | 162.0 | (148.9–182.8) | 152.2 | (141.8–161.0) | 0.009 | * |
| Weight | (kg) | 57.6 | (49.9–74.5) | 50.2 | (42.7–55.9) | 0.006 | * |
| BMI | (kg/m2) | 22.5 | (19.7–25.8) | 21.3 | (20.4–25.8) | N.S. | |
| | | | | | | | |
| T-bil | (mg/dL) | 3.3 | (0.5–19.6) | 0.8 | (0.4–3.6) | 0.040 | * |
| AST | (U/L) | 102 | (8–994) | 32 | (15–436) | N.S. | |
| ALT | (U/L) | 154 | (6–1342) | 36 | (9–210) | N.S. | |
| ALP | (U/L) | 1907 | (200–4541) | 371 | (139–774) | 0.010 | * |
| LDH | (U/L) | 192 | (117–673) | 198 | (156–473) | N.S. | |
| γ-GTP | (U/L) | 1440 | (29–1846) | 88 | (13–584) | 0.010 | * |
| WBC | (/μL) | 6530 | (3810–14600) | 4855 | (3970–14740) | N.S. | |
| CRP | (mg/dL) | 0.68 | (0.21–6.28) | 0.19 | (0–5.04) | N.S. | |
| | | | | | | | |
| CEA | (ng/mL) | 2.87 | (1.51–5.51) | 1.80 | (1.11–2.82) | 0.024 | * |
| CA19-9 | (U/mL) | 65.7 | (4.8–3746.1) | 7.6 | (5.1–17.1) | 0.010 | * |
| | | | | | | | |
| Tumor location | | | | | | | |
| Hilar/distal | | 4/4 | | | | | |
| TNM stage | | | | | | | |
| 1/2/3/4 | | 2/4/2/1 | | | | | |

Median (range)

N.S. not significant

Supplementary Table S3. All CCA-specific proteins

| | Accession number | Description | Gene name | P-value | Fold change |
|---|---|---|---|---|---|
| 1 | O15551 | Claudin-3 | CLDN3 | 0.000232 | 2.58 |
| 2 | Q9P2J5 | Leucine--tRNA ligase, cytoplasmic | LARS | 0.000233 | 3.29 |
| 3 | Q96CS3 | FAS-associated factor 2 | FAF2 | 0.000491 | 6.51 |
| 4 | Q9NX57 | Ras-related protein Rab-20 | RAB20 | 0.000721 | 21.48 |
| 5 | Q9Y678 | Coatomer subunit gamma-1 | COPG1 | 0.000825 | 2.75 |
| 6 | P04114 | Apolipoprotein B-100 | APOB | 0.001219 | 2.08 |
| 7 | P22694 | cAMP-dependent protein kinase catalytic subunit beta | PRKACB | 0.001835 | 2.51 |
| 8 | P62913 | 60S ribosomal protein L11 | RPL11 | 0.002573 | 8.13 |
| 9 | Q9HD43 | Receptor-type tyrosine-protein phosphatase H | PTPRH | 0.002640 | 2.93 |
| 10 | P02760 | Protein AMBP | AMBP | 0.002855 | 2.74 |
| 11 | Q86YQ8 | Copine-8 | CPNE8 | 0.003307 | 3.44 |
| 12 | Q6P6B1 | Glutamate-rich protein 5 | ERICH5 | 0.003354 | 10.02 |
| 13 | Q969L2 | Protein MAL2 | MAL2 | 0.003637 | 2.33 |
| 14 | Q14651 | Plastin-1 | PLS1 | 0.004065 | 5.18 |
| 15 | Q96TA1 | Niban-like protein 1 | FAM129B | 0.005052 | 2.66 |
| 16 | Q14451 | Growth factor receptor-bound protein 7 | GRB7 | 0.005113 | 4.72 |
| 17 | P00742 | Coagulation factor X | F10 | 0.005602 | 31.39 |
| 18 | Q4VNC1 | Probable cation-transporting ATPase 13A4 | ATP13A4 | 0.005734 | 2.89 |

| | | | | | |
|---|---|---|---|---|---|
| 19 | P09525 | Annexin A4 | ANXA4 | 0.005813 | 3.09 |
| 20 | P37059 | Estradiol 17-beta-dehydrogenase 2 | HSD17B2 | 0.005920 | 8.15 |
| 21 | P60903 | Protein S100-A10 | S100A10 | 0.006000 | 2.11 |
| 22 | P08865 | 40S ribosomal protein SA | RPSA | 0.006072 | 15.09 |
| 23 | Q9UHD8 | Septin-9 | SEPT9 | 0.006345 | 5.31 |
| 24 | H3BN98 | Uncharacterized protein (fragment) | | 0.006381 | 67.62 |
| 25 | P46776 | 60S ribosomal protein L27a | RPL27A | 0.006564 | 6.98 |
| 26 | Q8WUM4 | Programmed cell death 6-interacting protein | PDCD6IP | 0.006578 | 2.83 |
| 27 | Q15796 | Mothers against decapentaplegic homolog 2 | SMAD2 | 0.006663 | 5.91 |
| 28 | Q9BW30 | Tubulin polymerization-promoting protein family member 3 | TPPP3 | 0.006769 | 2.66 |
| 29 | P98196 | Probable phospholipid-transporting ATPase IH | ATP11A | 0.006838 | 2.33 |
| 30 | Q99816 | Tumor susceptibility gene 101 protein | TSG101 | 0.006861 | 3.35 |
| 31 | P19823 | Inter-alpha-trypsin inhibitor heavy chain H2 | ITIH2 | 0.007085 | 2.32 |
| 32 | Q06210 | Glutamine–fructose-6-phosphate aminotransferase [isomerizing] 1 | GFPT1 | 0.007092 | 5.77 |
| 33 | P34096 | Ribonuclease 4 | RNASE4 | 0.007147 | 4.23 |
| 34 | P68371 | Tubulin beta-4B chain | TUBB4B | 0.007213 | 2.74 |
| 35 | Q2M3M2 | Sodium/glucose cotransporter 4 | SLC5A9 | 0.007502 | 13.70 |
| 36 | Q9GZP0 | Platelet-derived growth factor D | PDGFD | 0.007548 | 9.26 |
| 37 | P78369 | Claudin-10 | CLDN10 | 0.007737 | 6.28 |
| 38 | P11021 | Endoplasmic reticulum chaperone BiP | HSPA5 | 0.008142 | 11.57 |
| 39 | Q13393 | Phospholipase D1 | PLD1 | 0.008517 | 2.56 |
| 40 | P11586 | C-1-tetrahydrofolate synthase, cytoplasmic | MTHFD1 | 0.008596 | 2.25 |

| | | | | | |
|---|---|---|---|---|---|
| 41 | Q717R9 | Cystin-1 | CYS1 | 0.008785 | 6.41 |
| 42 | Q9UK41 | Vacuolar protein sorting-associated protein 28 homolog | VPS28 | 0.008799 | 3.25 |
| 43 | Q9HD42 | Charged multivesicular body protein 1a | CHMP1A | 0.008976 | 7.50 |
| 44 | P05787 | Keratin, type II cytoskeletal 8 | KRT8 | 0.009257 | 3.58 |
| 45 | Q9NZZ3 | Charged multivesicular body protein 5 | CHMP5 | 0.009466 | 2.91 |
| 46 | P98160 | Basement membrane-specific heparan sulfate proteoglycan core protein | HSPG2 | 0.009573 | 3.05 |
| 47 | P13611 | Versican core protein | VCAN | 0.009820 | 6.69 |
| 48 | Q14204 | Cytoplasmic dynein 1 heavy chain 1 | DYNC1H1 | 0.010018 | 2.84 |
| 49 | Q9H0E2 | Toll-interacting protein | TOLLIP | 0.010042 | 7.44 |
| 50 | Q9BQE3 | Tubulin alpha-1C chain | TUBA1C | 0.010217 | 2.17 |
| 51 | P39060 | Collagen alpha-1(XVIII) chain | COL18A1 | 0.010323 | 7.26 |
| 52 | Q08257 | Quinone oxidoreductase | CRYZ | 0.010877 | 3.32 |
| 53 | Q9NP79 | Vacuolar protein sorting-associated protein VTA1 homolog | VTA1 | 0.010888 | 8.96 |
| 54 | Q9BVA1 | Tubulin beta-2B chain | TUBB2B | 0.010895 | 15.02 |
| 55 | P07437 | Tubulin beta chain | TUBB | 0.011092 | 3.02 |
| 56 | P05023 | Sodium/potassium-transporting ATPase subunit alpha-1 | ATP1A1 | 0.011095 | 4.42 |
| 57 | P50914 | 60S ribosomal protein L14 | RPL14 | 0.011483 | 4.20 |
| 58 | O75608 | Acyl-protein thioesterase 1 | LYPLA1 | 0.011817 | 5.17 |
| 59 | Q14108 | Lysosome membrane protein 2 | SCARB2 | 0.011822 | 2.36 |
| 60 | P62847 | 40S ribosomal protein S24 | RPS24 | 0.011882 | 4.26 |
| 61 | P10909 | Clusterin | CLU | 0.012077 | 3.98 |
| 62 | O14979 | Heterogeneous nuclear ribonucleoprotein D-like | HNRNPDL | 0.012158 | 4.13 |

| | | | | | |
|---|---|---|---|---|---|
| 63 | Q86XT2 | Vacuolar protein sorting-associated protein 37D | VPS37D | 0.013068 | 7.71 |
| 64 | Q9H4G0 | Band 4.1-like protein 1 | EPB41L1 | 0.013423 | 7.78 |
| 65 | Q9GZM7 | Tubulointerstitial nephritis antigen-like | TINAGL1 | 0.015175 | 6.29 |
| 66 | Q01813 | ATP-dependent 6-phosphofructokinase, platelet type | PFKP | 0.015896 | 4.36 |
| 67 | P62879 | Guanine nucleotide-binding protein G(I)/G(S)/G(T) subunit beta-2 | GNB2 | 0.015909 | 2.10 |
| 68 | Q9Y6W3 | Calpain-7 | CAPN7 | 0.015976 | 3.18 |
| 69 | Q3B8N2 | Galectin-9B | LGALS9B | 0.016036 | 4.95 |
| 70 | Q9NYS7 | WD repeat and SOCS box-containing protein 2 | WSB2 | 0.016491 | 2.20 |
| 71 | P36955 | Pigment epithelium-derived factor | SERPINF1 | 0.017125 | 3.39 |
| 72 | Q14435 | Polypeptide N-acetylgalactosaminyltransferase 3 | GALNT3 | 0.017145 | 16.51 |
| 73 | P12236 | ADP/ATP translocase 3 | SLC25A6 | 0.017950 | 4.47 |
| 74 | Q9Y2T3 | Guanine deaminase | GDA | 0.018061 | 12.02 |
| 75 | P02747 | Complement C1q subcomponent subunit C | C1QC | 0.018073 | 2.62 |
| 76 | O43520 | Phospholipid-transporting ATPase IC | ATP8B1 | 0.018216 | 3.10 |
| 77 | O60658 | High-affinity cAMP-specific and IBMX-insensitive 3',5'-cyclic phosphodiesterase 8A | PDE8A | 0.018597 | 3.17 |
| 78 | P26572 | Alpha-1,3-mannosyl-glycoprotein 2-beta-N-acetylglucosaminyltransferase | MGAT1 | 0.019067 | 6.67 |
| 79 | Q99747 | Gamma-soluble NSF attachment protein | NAPG | 0.019490 | 9.35 |
| 80 | O75351 | Vacuolar protein sorting-associated protein 4B | VPS4B | 0.019602 | 3.14 |
| 81 | P62829 | 60S ribosomal protein L23 | RPL23 | 0.019631 | 3.37 |
| 82 | P51178 | 1-PhosphatidylInositol 4,5-bisphosphate phosphodiesterase delta-1 | PLCD1 | 0.019640 | 4.63 |
| 83 | Q5VW32 | BRO1 domain-containing protein BROX | BROX | 0.019844 | 2.56 |
| 84 | O15438 | Canalicular multispecific organic anion transporter 2 | ABCC3 | 0.020068 | 4.30 |

| | | | | | |
|---|---|---|---|---|---|
| 85 | Q16629 | Serine/arginine-rich splicing factor 7 | SRSF7 | 0.020626 | 14.28 |
| 86 | Q16555 | Dihydropyrimidinase-related protein 2 | DPYSL2 | 0.020749 | 9.03 |
| 87 | P55056 | Apolipoprotein C-IV | APOC4 | 0.021059 | 8.87 |
| 88 | P62241 | 40S ribosomal protein S8 | RPS8 | 0.021239 | 2.55 |
| 89 | Q13557 | Calcium/calmodulin-dependent protein kinase type II subunit delta | CAMK2D | 0.021345 | 5.89 |
| 90 | Q13418 | Integrin-linked protein kinase | ILK | 0.021600 | 3.86 |
| 91 | P84077 | ADP-ribosylation factor 1 | ARF1 | 0.021632 | 5.40 |
| 92 | Q9Y2A9 | N-Acetyllactosaminide beta-1,3-N-acetylglucosaminyltransferase 3 | B3GNT3 | 0.021897 | 9.32 |
| 93 | P13866 | Sodium/glucose cotransporter 1 | SLC5A1 | 0.022042 | 2.09 |
| 94 | Q00610 | Clathrin heavy chain 1 | CLTC | 0.022530 | 2.44 |
| 95 | P27216 | Annexin A13 | ANXA13 | 0.022902 | 3.12 |
| 96 | P78417 | Glutathione S-transferase omega-1 | GSTO1 | 0.022956 | 9.36 |
| 97 | P16422 | Epithelial cell adhesion molecule | EPCAM | 0.023022 | 13.02 |
| 98 | P36405 | ADP-ribosylation factor-like protein 3 | ARL3 | 0.023568 | 5.58 |
| 99 | P42330 | Aldo-keto reductase family 1 member C3 | AKR1C3 | 0.023765 | 2.53 |
| 100 | Q14520 | Hyaluronan-binding protein 2 | HABP2 | 0.024753 | 2.79 |
| 101 | Q14376 | UDP-glucose 4-epimerase | GALE | 0.025093 | 4.25 |
| 102 | P20337 | Ras-related protein Rab-3B | RAB3B | 0.025594 | 2.90 |
| 103 | O14672 | Disintegrin and metalloproteinase domain-containing protein 10 | ADAM10 | 0.026215 | 5.93 |
| 104 | P05026 | Sodium/potassium-transporting ATPase subunit beta-1 | ATP1B1 | 0.026245 | 2.53 |
| 105 | O14950 | Myosin regulatory light chain 12B | MYL12B | 0.026341 | 5.31 |
| 106 | Q14116 | Interleukin-18 | IL18 | 0.026552 | 2.81 |

| | | | | | |
|---|---|---|---|---|---|
| 107 | Q9H9H4 | Vacuolar protein sorting-associated protein 37B | VPS37B | 0.026652 | 2.95 |
| 108 | P00352 | Retinal dehydrogenase 1 | ALDH1A1 | 0.027009 | 2.01 |
| 109 | O43633 | Charged multivesicular body protein 2a | CHMP2A | 0.027210 | 3.21 |
| 110 | Q8TE68 | Epidermal growth factor receptor kinase substrate 8-like protein 1 | EPS8L1 | 0.028753 | 2.10 |
| 111 | P15291 | Beta-1,4-galactosyltransferase 1 | B4GALT1 | 0.029258 | 3.08 |
| 112 | P30085 | UMP-CMP kinase | CMPK1 | 0.029344 | 2.05 |
| 113 | Q15366 | Poly(rC)-binding protein 2 | PCBP2 | 0.029939 | 2.25 |
| 114 | P20339 | Ras-related protein Rab-5A | RAB5A | 0.030110 | 4.17 |
| 115 | Q15181 | Inorganic pyrophosphatase | PPA1 | 0.030279 | 2.20 |
| 116 | Q9Y224 | RNA transcription, translation and transport factor protein | RTRAF | 0.031267 | 3.41 |
| 117 | P08754 | Guanine nucleotide-binding protein G(k) subunit alpha | GNAI3 | 0.031363 | 2.42 |
| 118 | Q6PCB0 | von Willebrand factor A domain-containing protein 1 | VWA1 | 0.031511 | 3.29 |
| 119 | Q12797 | Aspartyl/asparaginyl beta-hydroxylase | ASPH | 0.031742 | 3.48 |
| 120 | Q9P0V3 | SH3 domain-binding protein 4 | SH3BP4 | 0.032538 | 3.85 |
| 121 | Q5XXA6 | Anoctamin-1 | ANO1 | 0.032636 | 2.13 |
| 122 | P53990 | IST1 homolog | IST1 | 0.032639 | 2.12 |
| 123 | O95471 | Claudin-7 | CLDN7 | 0.032680 | 8.21 |
| 124 | P14866 | Heterogeneous nuclear ribonucleoprotein L | HNRNPL | 0.033079 | 6.48 |
| 125 | Q05209 | Tyrosine-protein phosphatase non-receptor type 12 | PTPN12 | 0.033402 | 2.25 |
| 126 | Q9UBI6 | Guanine nucleotide-binding protein G(I)/G(S)/G(O) subunit gamma-12 | GNG12 | 0.033635 | 3.32 |
| 127 | Q8ND76 | Cyclin-Y | CCNY | 0.034088 | 2.57 |
| 128 | P08183 | Multidrug resistance protein 1 | ABCB1 | 0.034098 | 3.43 |

| | | | | | |
|---|---|---|---|---|---|
| 129 | O94826 | Mitochondrial import receptor subunit TOM70 | TOMM70 | 0.034329 | 3.31 |
| 130 | P31943 | Heterogeneous nuclear ribonucleoprotein H | HNRNPH1 | 0.034648 | 2.89 |
| 131 | Q9BQL6 | Fermitin family homolog 1 | FERMT1 | 0.034820 | 2.19 |
| 132 | P05162 | Galectin-2 | LGALS2 | 0.035133 | 5.26 |
| 133 | Q93052 | Lipoma-preferred partner | LPP | 0.035138 | 4.50 |
| 134 | P52895 | Aldo-keto reductase family 1 member C2 | AKR1C2 | 0.035570 | 2.77 |
| 135 | Q99805 | Transmembrane 9 superfamily member 2 | TM9SF2 | 0.035677 | 8.00 |
| 136 | Q07960 | Rho GTPase-activating protein 1 | ARHGAP1 | 0.035950 | 5.26 |
| 137 | O43396 | Thioredoxin-like protein 1 | TXNL1 | 0.037047 | 4.72 |
| 138 | P49411 | Elongation factor Tu, mitochondrial | TUFM | 0.037328 | 7.82 |
| 139 | Q16650 | T-box brain protein 1 | TBR1 | 0.037824 | 3.42 |
| 140 | Q71U36 | Tubulin alpha-1A chain | TUBA1A | 0.038011 | 10.87 |
| 141 | Q7KZF4 | Staphylococcal nuclease domain-containing protein 1 | SND1 | 0.038245 | 4.51 |
| 142 | P02649 | Apolipoprotein E | APOE | 0.038489 | 3.30 |
| 143 | P83731 | 60S ribosomal protein L24 | RPL24 | 0.038670 | 13.28 |
| 144 | Q8TAD7 | Overexpressed in colon carcinoma 1 protein | OCC1 | 0.038839 | 47.32 |
| 145 | P62424 | 60S ribosomal protein L7a | RPL7A | 0.040084 | 4.40 |
| 146 | P40925 | Malate dehydrogenase, cytoplasmic | MDH1 | 0.040101 | 20.03 |
| 147 | O00159 | Unconventional myosin-Ic | MYO1C | 0.040211 | 2.63 |
| 148 | Q9UP52 | Transferrin receptor protein 2 | TFR2 | 0.040977 | 3.91 |
| 149 | O94832 | Unconventional myosin-Id | MYO1D | 0.041215 | 2.19 |
| 150 | P27635 | 60S ribosomal protein L10 | RPL10 | 0.041502 | 3.28 |

| | | | | | |
|---|---|---|---|---|---|
| 151 | P10646 | Tissue factor pathway inhibitor | TFPI | 0.041861 | 3.89 |
| 152 | P62266 | 40S ribosomal protein S23 | RPS23 | 0.042762 | 4.63 |
| 153 | Q9C0K7 | STE20-related kinase adapter protein beta | STRADB | 0.043303 | 4.18 |
| 154 | Q92599 | Septin-8 | 8-Sep | 0.043528 | 7.52 |
| 155 | P53621 | Coatomer subunit alpha | COPA | 0.043862 | 2.48 |
| 156 | Q9BUF5 | Tubulin beta-6 chain | TUBB6 | 0.043887 | 2.09 |
| 157 | P11172 | Uridine 5'-monophosphate synthase | UMPS | 0.043888 | 5.62 |
| 158 | Q9GZU7 | Carboxy-terminal domain RNA polymerase II polypeptide A small phosphatase 1 | CTDSP1 | 0.044412 | 13.31 |
| 159 | Q8TBF2 | Prostamide/prostaglandin F synthase | PRXL2B | 0.044498 | 2.39 |
| 160 | Q9UHG0 | Doublecortin domain-containing protein 2 | DCDC2 | 0.045061 | 2.90 |
| 161 | P11182 | Lipoamide acyltransferase component of branched-chain alpha-keto acid dehydrogenase complex, mitochondrial | DBT | 0.045542 | 2.38 |
| 162 | P62851 | 40S ribosomal protein S25 | RPS25 | 0.047421 | 10.04 |
| 163 | Q9Y625 | Glypican-6 | GPC6 | 0.047553 | 4.88 |
| 164 | P18124 | 60S ribosomal protein L7 | RPL7 | 0.047780 | 3.11 |
| 165 | P39023 | 60S ribosomal protein L3 | RPL3 | 0.047995 | 4.56 |
| 166 | O60513 | Beta-1,4-galactosyltransferase 4 | B4GALT4 | 0.048659 | 13.28 |

Supplementary Table S4. Comparison of CLDN3 by immunohistochemical staining

| | Case 1 | Case 2 | Case 3 | Case 4 | Case 5 | P-value | |
|---|---|---|---|---|---|---|---|
| Normal | 1 (1,1) | 0 (0,0) | 1 (0,0) | 4 (2,2) | 2 (1,2) | 0.0153 | * |
| CCA | 8 (2,4) | 9 (3,3) | 4 (2,2) | 9 (3,3) | 12 (3,4) | | |

IHC score (intensity, positive percentage)

Normal, normal bile duct epithelium

CCA, cholangiocarcinoma
